# Chlorophyll Rings around Ocean Eddies in the North Pacific

**DOI:** 10.1038/s41598-018-38457-8

**Published:** 2019-02-14

**Authors:** Guangjun Xu, Changming Dong, Yu Liu, Peter Gaube, Jingsong Yang

**Affiliations:** 1 Oceanic Modeling and Observation Laboratory, Nanjing University of Information Science and Technology, Nanjing, 210044 China; 2 State Key Laboratory of Satellite Ocean Environment Dynamics, Second Institute of Oceanography, MNR, Hangzhou, 310012 China; 3 Department of Atmospheric and Oceanic Sciences, University of California, Los Angeles, CA 90095 USA; 4 Department of Air-Sea Interaction and Remote Sensing, Applied Physics Laboratory, Seattle, WA 98028 USA

## Abstract

Chlorophyll rings (CRs) are defined as elevated chlorophyll along eddy peripheries and have been observed in anticyclonic oceanic eddies occasionally. This study presents observations of CRs around both anticyclonic and cyclonic eddies from a large observational data set. An innovative algorithm is developed to identify CRs from satellite observations of sea level anomalies and near-surface chlorophyll concentration in the North Pacific Ocean between 2003 and 2010. The results show that only 1% of mesoscale eddies are associated with CRs, which implies the CRs are not ubiquitous. We propose two potential generation mechanisms for CRs: horizontal advection and wind-current interaction. The former dominates the formation of about two-thirds of the CRs. The CRs associated with both cyclones and anticyclones represents an important contribution to better understanding of mesoscale physical/biological coupled phenomena.

## Introduction

Eddies influence marine phytoplankton via a myriad of mechanisms including: (i) eddy pumping resulting from vertical displacement of isopycnals leading to vertical transports in eddy cores^1–6^; (ii) eddy stirring along their peripheries^2–5,7,8^; (iii) trapping of ecosystems during the formation of eddies^2,3,9,10^; (iv) eddy-induced Ekman pumping associated with spatial variations in wind stress^2,3,11–13^; (v) ageostrophic upwelling at submesoscale^11,14,15^.

To date, CRs have only been observed in isolated incidents along the peripheries of anticyclonic eddies in regions such as the Bering Sea^16^, the Southern Antarctic Circumpolar Current Front^17^, the Pagasitikos Gulf^18^, the lee of Hawaii Islands^19^ and the South China Sea^20^. Mechanisms that have been proposed for CRs around anticyclones include lateral advection of water with high concentration from nearby regions^3^, the upwelling of nutrient-rich water along the tilting isopycnals around the periphery of anticyclonic eddies^16–18^, vertical jet of nutrients in the regions of strong stretching of eddies^19^, and the outwards radial displacements of phytoplankton as a result of radial momentum imbalances along eddy peripheries^20^. In the present study, we develop an algorithm to automatically detect CRs from a large dataset in the North Pacific Ocean (5°N to 65°N and 100°E to 75°W), where 241,380 individual eddy realizations are identified, and investigate whether the CRs can take place along both anticyclonic and cyclonic eddies. Finally, we propose potential mechanisms for the CRs generation.

## Results

The study is primarily based on chlorophyll concentration data and sea surface geostrophic currents velocities which are used to identify CRs. In addition, sea surface temperature (SST), and surface vector winds are also employed in the discussions of the potential mechanisms for CRs.

A total of 121,846 cyclonic and 119,534 anticyclonic realizations with lifetimes greater than 4 weeks are matched with concurrent chlorophyll concentration data between 2003 and 2010. Each of these realizations meets the criteria of more than 30% chlorophyll concentration coverage within the region inscribed by a circle whose radius is 2.5 times that of the eddy. Based on the criteria for CRs identification (see Methods for details), 1,286 cyclonic eddies and 1,506 anticyclonic eddies are associated with CRs. These account for about 1% of all the eddies detected. Anticyclones have a 17% higher likelihood of being surrounded by a ring when compared to cyclones.

**Figure 1:**
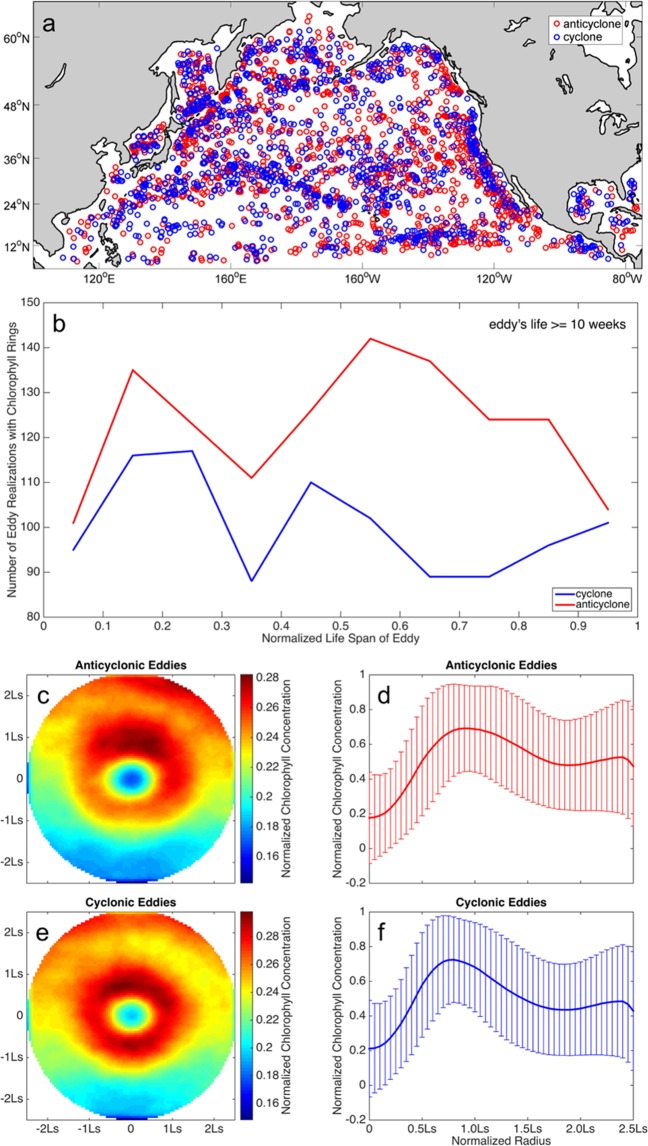
Statistical characteristics of CRs. (**a**) Spatial distributions of eddies with CRs. (**b**) Number of eddy realizations with CRs vs normalized life span of eddy. (**c**) Ensemble averaged normalized chlorophyll concentration of the CRs around anticyclones. (**d)** Averaged normalized chlorophyll as a function of the eddy normalized radius with error bars in anticyclones. Error bars refer to the standard deviations. (**e**,**f**) same as (**c**,**d**) expect for the cyclonic eddy. Figures are plotted using MATLAB R2014b (http://www.mathworks.com) with M_Map (a mapping package, http://www.eos.ubc.ca/~rich/map.html).

To quantify the significance of CRs at a basin scale, area-integrated chlorophyll concentrations are calculated both over the area of all CRs and over the total area of the North Pacific basin over one climatological year. The averaged chlorophyll concentration over the unit area within CRs is 0.47 ± 0.07 mg m^−3^, which is about twice of that over the whole North Pacific basin, 0.23 ± 0.01 mg m^−3^. Considering that the total area covered by CRs is about 9.28 × 10^6^ km^2^ over one climatological year, and that the total area of the whole North Pacific basin is 7.20 × 10^7^ km^2^, CRs carry approximately 25% of the surface chlorophyll content at the basin scale. There are more CRs at the eastern and western boundaries as well as the Kuroshio extension (Fig. 1a). These regions are characterized by either high chlorophyll concentration (east and west boundary) or large eddy kinetic energy (Kuroshio extension).

The lifetime of an individual eddy is separated into several stages, defined as proportion of total lifetime (ranging from 0 to 1)^2,21^: formation stage (0~0.1), intensification stage (0.1~0.3), mature stage (0.3~0.8) and aged stage (0.8~1). Specific life stages are counted for CRs within eddies, whose lifespans are longer than 10 weeks. This analysis reveals that CRs in eddies of both polarities are most common during the intensification and mature stage (Fig. 1b). In anticyclones, however, CRs occur more frequently in the late mature stage than that during eddy intensification. In contrast, CRs develop more frequently during eddy intensification than mature stage in cyclones.

Spatial distributions of the ensemble mean chlorophyll concentration of CRs around anticyclonic eddies and cyclonic eddies are shown in Fig. 1c,e. Normalized chlorophyll concentrations are presented in the figures, which are relative to the maximum chlorophyll concentration within each CRs. Averaged normalized chlorophyll concentrations are shown as a function of the eddy radius in Fig. 1d,f.

## Discussion

Two potential mechanisms are proposed for the CR generation: horizontal advection and wind-current interaction.

**Figure 2:**
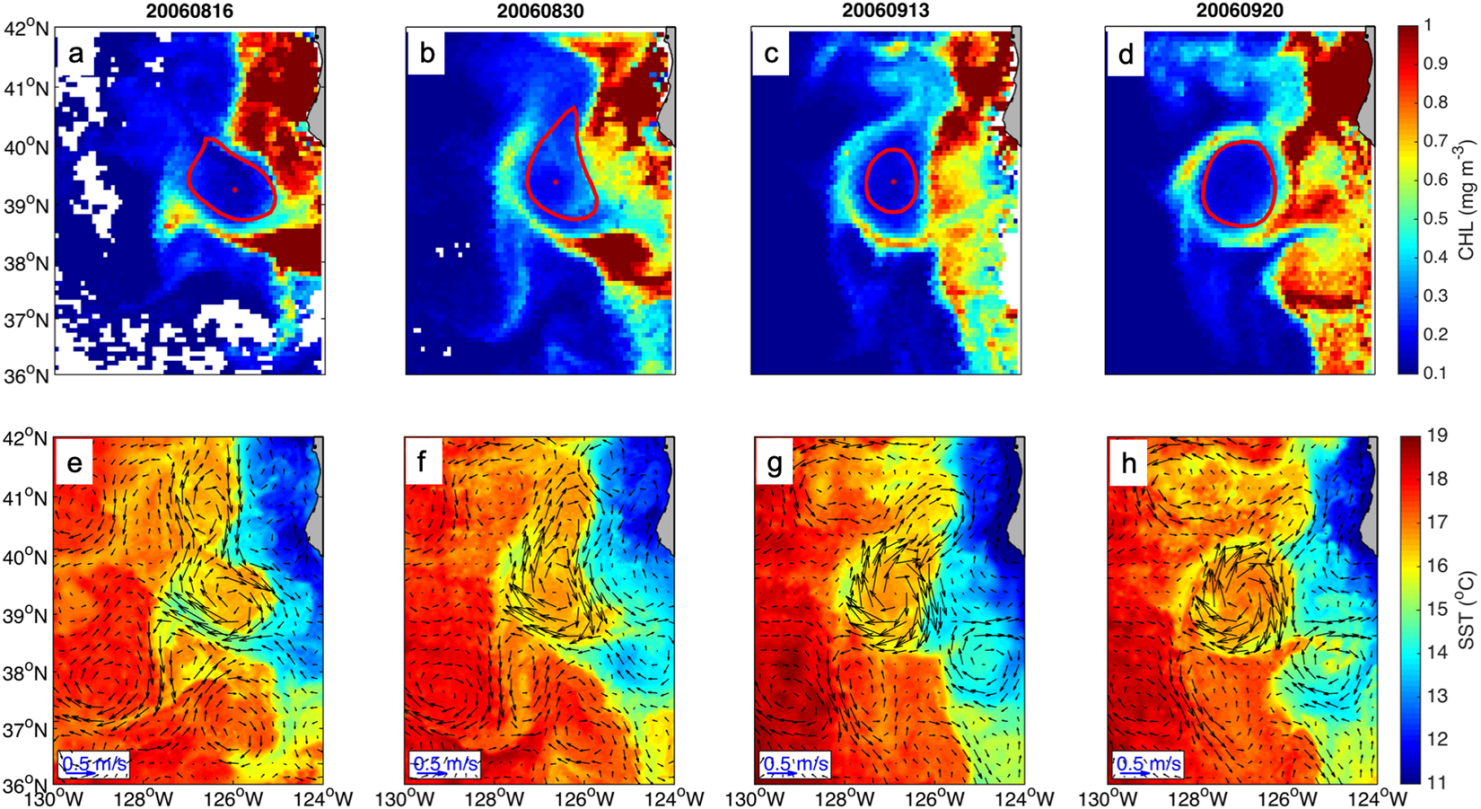
An anticyclonic eddy generated at the east coast of Pacific during the period from Aug 16 to Sep 20, 2006. Chlorophyll is advected from the coastal region to the periphery of the eddy (**a**–**d**). SST and current velocity fields show the same pattern (**e**–**h**). Red solid line represents the eddy edge. Figures are plotted using MATLAB R2014b (http://www.mathworks.com) with M_Map (a mapping package, http://www.eos.ubc.ca/~rich/map.html).

(1) Horizontal advection of chlorophyll in the ambient water of eddies is reported to be one of the dominant mechanisms by which eddies influence chlorophyll distribution^2,7^. The generation of CRs by horizontal advection can be seen in the example shown in Fig. 2a~d where an anticyclonic eddy in the eastern boundary of the North Pacific is observed to generate a CR. The rotating flow of the eddy results in a chlorophyll “tongue” south of the eddy on Aug 16, 2006. The chlorophyll tongue moves to the north with the development of eddy. Eventually higher chlorophyll concentration appears as a ring structure. The chlorophyll concentration within the region 0.5 Ls~1.5 Ls of the anticyclonic eddy is enhanced by 0.12~0.21 mg m^−3^ during the formation, relative to the climatological value. The SST shows additional evidence of horizontal advection (Fig. 2e~h). A cold tongue propagates from the shore to the offshore while the anticyclonic eddy rotates. Consequently, a ring structure with cold water is developed along the periphery of the eddy. The anticyclonic eddy survives for 44 weeks and the analysis of the evolution of the CRs suggests that the ring occurs at a normalized lifetime of 0.2~0.35, defined here as the eddy intensification stage.

**Figure 3:**
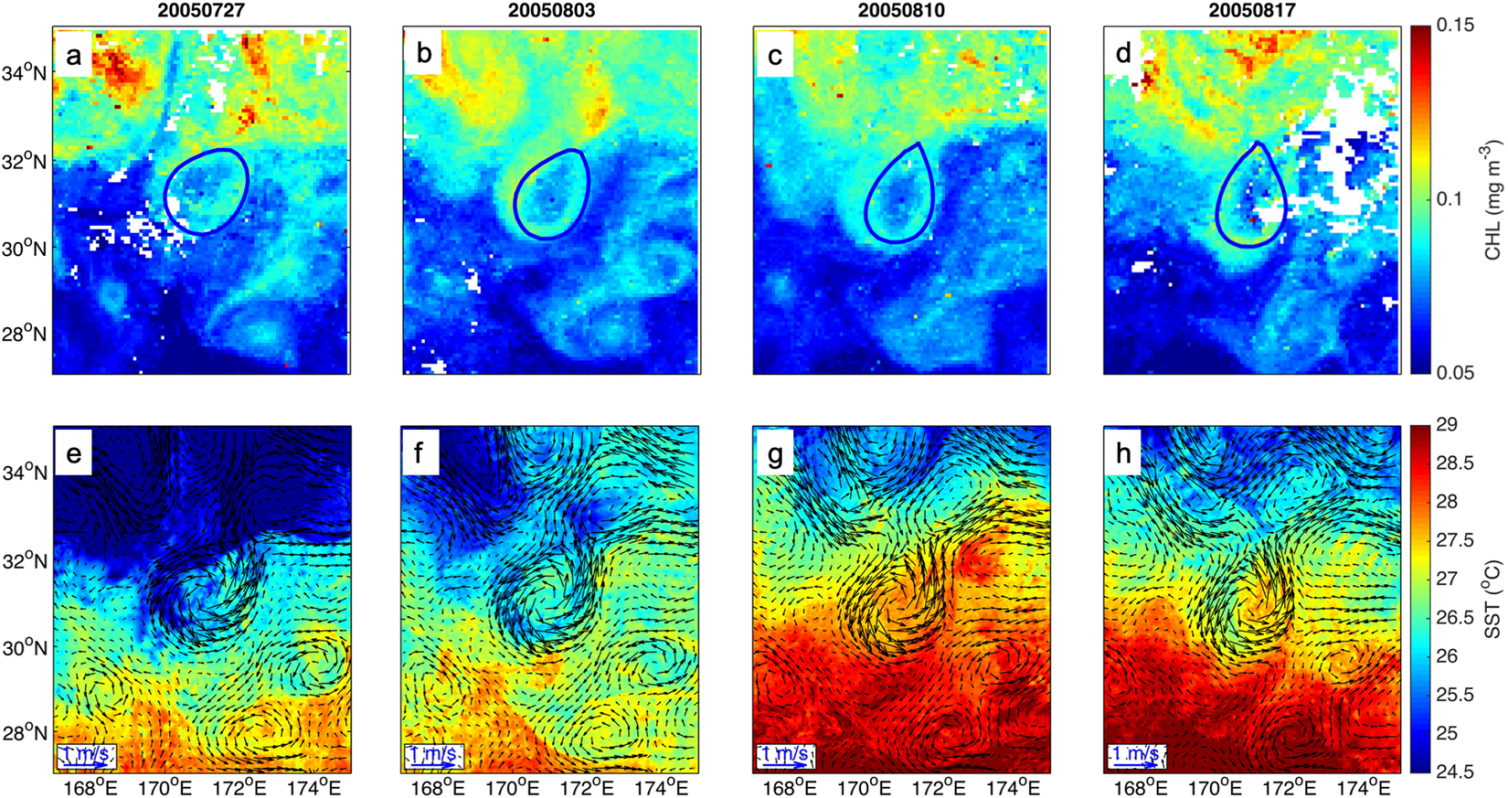
As in Fig. 2 but for cyclonic eddy generated at the boundary of the Extension of Kuroshio during the period from Jul 27 to Aug 17, 2005. Blue solid line represents the eddy edge. Figures are plotted using MATLAB R2014b (http://www.mathworks.com) with M_Map (a mapping package, http://www.eos.ubc.ca/~rich/map.html).

Analogously, a CR is formed around a cyclonic eddy at the boundary of the Extension of Kuroshio in Fig. 3a~d. The local chlorophyll concentrations within this CR increase by about 0.01 mg m^−3^. In SST images (Fig. 3e~h), a cold tongue moves southwards while the rotation of cyclonic eddy on Jul 27, 2005, resulting in a closed circle along the eddy periphery. A CR appears during the eddy intensification stage (0.46~0.54) in the life span of 65 weeks.

Comparing the averaged chlorophyll concentration along the eddies’ peripheries (between 0.5 Ls and 1.5 Ls) and outside the eddies (greater than 1.5 Ls), we distinguish whether CRs are generated by the horizontal transport of chlorophyll. If the averaged chlorophyll concentration outside the eddies is higher than that within a CR along the periphery, the CR is judged to be associated with the contribution of horizontal advection. 2123 CRs (about 66%) are found to be formed by this mechanism.

(2) Wind-current interaction via eddy-induced Ekman pumping could generate vertical transport, possibly resulting in CRs observed around eddies. In regions of intense wind stress and relatively strong horizontal current gradients (e.g., at the edges of mesoscale features), Ekman-induced vertical velocities are generated not only by the curl of the wind stress but also by the interaction of the surface wind stress with the geostrophic relative vorticity gradient^19,22–25^. With the Ekman transport modified by the surface geostrophic vorticity ${\zeta }_{g}$, the total eddy-induced Ekman pumping *W*_*tot*_ is

1 $$\begin{array}{rcl}{W}_{tot} & = & \frac{1}{{\rho }_{0}}\nabla \times [\frac{\mathop{\tau }\limits^{\rightharpoonup }}{(f+{\zeta }_{g})}]\\  & \approx  & \mathop{\underbrace{\frac{\nabla \times \mathop{\tau }\limits^{\rightharpoonup }}{{\rho }_{0}(f+{\zeta }_{g})}}}\limits_{{W}_{c}}+\mathop{\underbrace{\frac{1}{{\rho }_{0}{(f+{\zeta }_{g})}^{2}}({\tau }_{x}\frac{\partial {\zeta }_{g}}{\partial y}-{\tau }_{y}\frac{\partial {\zeta }_{g}}{\partial x})}}\limits_{{W}_{\zeta }}\end{array}$$

where ${\zeta }_{g}$ is the vertical component of the geostrophic relative vorticity, ${\rho }_{0}$=1020 kg m^−3^ is the (assumed constant) density of sea surface water, $f$ is the Coriolis parameter, ${\tau }_{x}$ and ${\tau }_{y}$ are zonal and meridional components of the surface wind stress $\mathop{\tau }\limits^{\rightharpoonup }$, respectively. Small magnitude of the meridional derivative of the Coriolis parameter contribution to the total Ekman pumping in (1) is ignored. Surface stress curl-induced Ekman pumping, ${{\rm{W}}}_{{\rm{c}}}$ (the first term on the right side of (1)), is determined by the wind tress curl because that $f+{\zeta }_{g}$ in the expression of ${W}_{{\rm{c}}}$ is always positive for mesoscale eddies: when the wind stress curl is positive (negative), it generates the upwelling (downwelling). Vorticity gradient-induced Ekman pumping, ${W}_{\zeta }$ (the second term on the right side of (1)), generates a dipole of Ekman upwelling and downwelling within the interior of ocean eddies.

**Figure 4:**
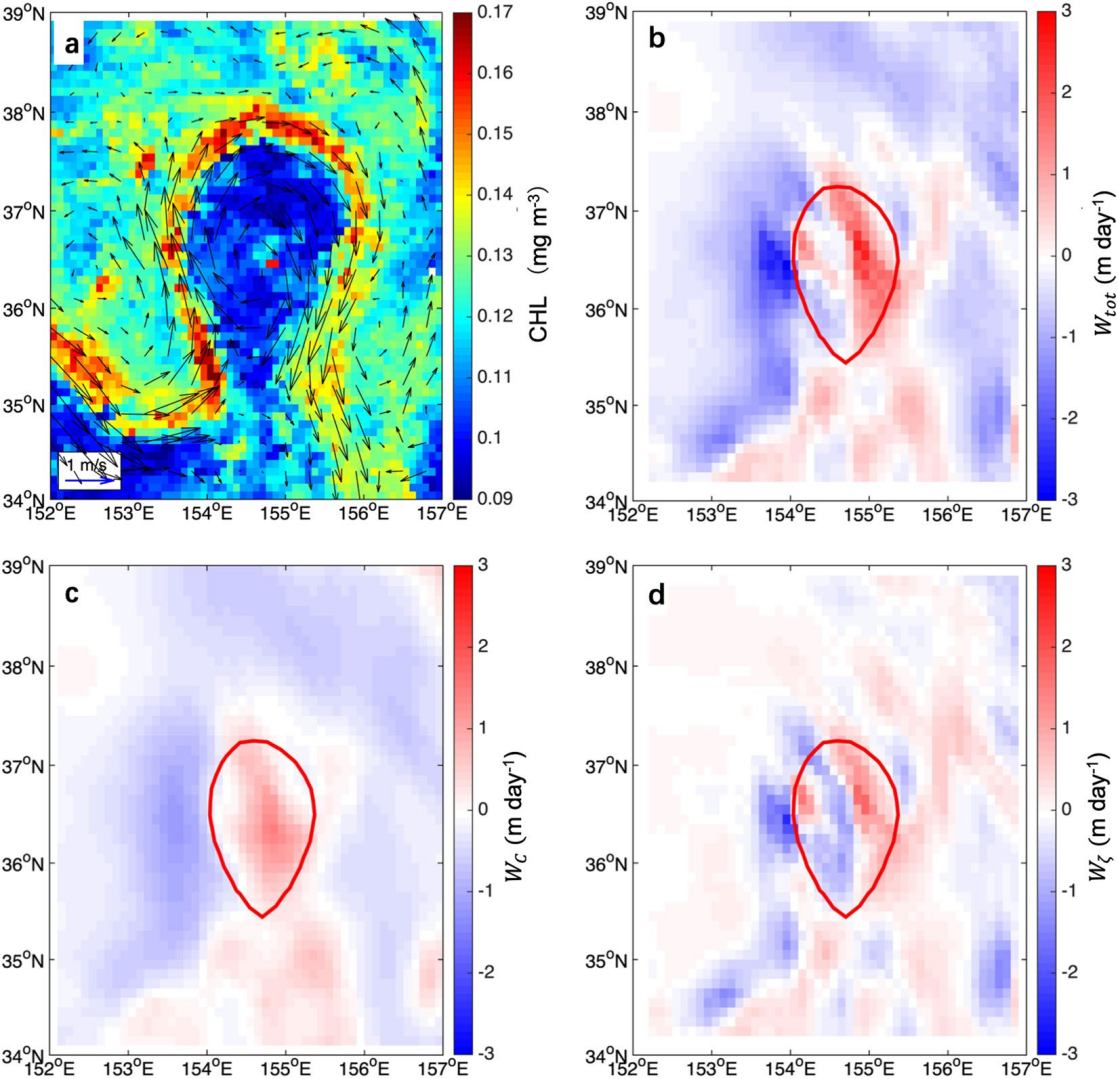
Eddy-induced Ekman pumping in the anticyclonic eddy on Aug 31, 2005. (**a**) A CR in anticyclonic eddy on Aug 31, 2005. The color denotes chlorophyll concentration, and the vectors are the current fields at the sea surface. (**b**) Total Ekman pumping, ${W}_{tot}$. (**c**) Surface stress curl–induced Ekman pumping, ${W}_{C}$. (**d**) Vorticity gradient–induced Ekman pumping, ${W}_{\zeta }$. Red solid line represents the eddy edge. Figures are plotted using MATLAB R2014b (http://www.mathworks.com) with M_Map (a mapping package, http://www.eos.ubc.ca/~rich/map.html).

Figure 4a shows a CR in the anticyclonic eddy on Aug 31, 2005. The interaction of intense wind stress with sea surface currents results in upward vertical transport (1.5 m day^−1^) in the east part of the eddy (Fig. 4b). The portion resulting from the curl of the surface stress, in Fig. 4c, generates upwelling within the anticyclone, while the contribution resulting the interaction of the surface stress with surface current vorticity gradient induces a dipole of upwelling and downwelling in the eddy, in Fig. 4d.

**Figure 5:**
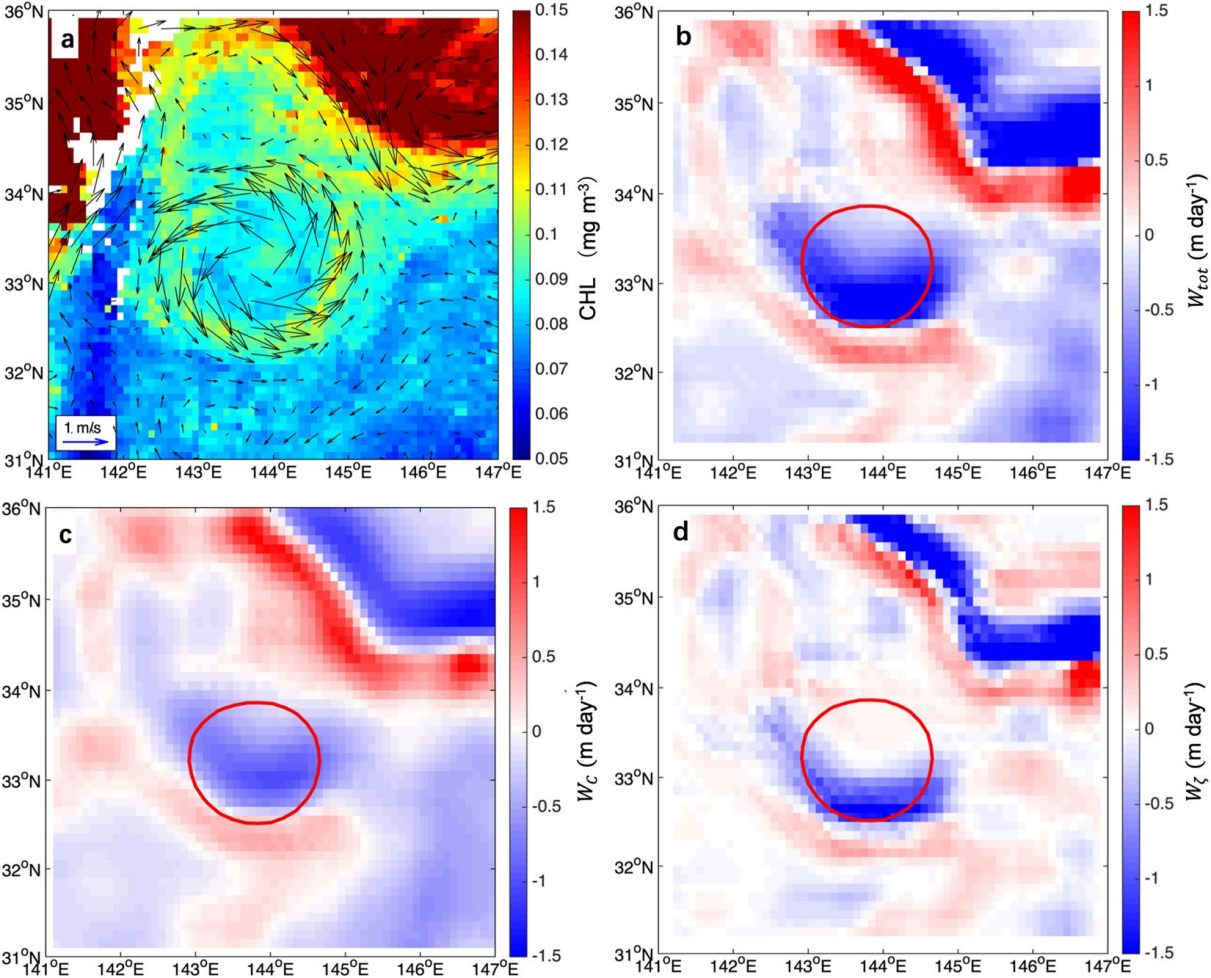
As in Fig. 4 but for a cyclonic eddy on Aug 03, 2005. Blue solid line represents the eddy edge. Figures are plotted using MATLAB R2014b (http://www.mathworks.com) with M_Map (a mapping package, http://www.eos.ubc.ca/~rich/map.html).

Similarly, eddy-induced Ekman pumping could also generate along the periphery of cyclonic eddy (Fig. 5). Upward vertical transport (0.6 m day^−1^) resulting from the total Ekman pumping appeares in the southeastern side of eddy edge. Additionally, the eddy is located south of a high-chlorophyll region, hence horizontal advection can also play a role in the CR formation observed.

This is the first endeavor to investigate CRs systematically. It shows that the CRs can occur in both cyclonic and anticyclonic eddies. An innovative algorithm to identify CRs from a large set of eddies is developed. CRs are more likely to be observed along the periphery of anticyclones when compared to cyclones. Two potential CR generation mechanisms are proposed: horizontal advection which transports the chlorophyll from adjacent areas; while vertical transports caused by eddy-induced Ekman pumping can bring nutrient and phytoplankton into the surface layer. Satellite observations and a reanalysis numerical product are applied to explore mechanisms that form observed CRs. We found that horizontal advection explains the formation of about two-thirds of CRs identified in the North Pacific.

Two potential mechanisms are proposed in this study, however, there are also various mechanisms of vertical transport of nutrients, chlorophyll and phytoplankton below the surface, which might be at play at any given time, for examples: local upwelling at submesoscale induced by symmetric instability could bring waters rich in nutrients into the euphotic layer^15,26^; internal waves could also uplift isopycnals high in nutrients within eddies^27^; ageostrophic secondary circulation could contribute to the vertical transport upward and downward as well^25,27–30^. Currently, there is still a challenge as the spatial-temporal resolution of observations is not available to resolve the submesoscale processes. Because of the interdependencies among various processes and scales, it is difficult to ascribe the vertical advection nutrients to individual mechanisms^30^, which requires further study in future.

## Methods

### Observational Data

The data used in the present study include several observational data: satellite remote sensing ocean color data (chlorophyll concentration), sea surface geostrophic currents velocities, sea surface temperature and wind vectors.

Eddies identification is based on weekly-mean sea surface geostrophic currents with a spatial resolution of 1/4°×1/4° which are obtained from AVISO (www.aviso.oceanobs.com). An automated algorithm based on the geometry of the velocity vectors of the flow field^31^ is used to identify and track mesoscale eddies. Our research is based on eddies with lifetimes of four weeks and longer.

Chlorophyll concentration data used in this paper are downloaded from http://wiki.icess.ucsb.edu/measures/index.php/GSM. It is a merged product of SeaWiFS, Meris, and MODIS-Aqua with a resolution of 9 km every 8 days, generated using the Garver-Siegel-Maritorena (GSM) model^32,33^.

SST data are used to illustrate the developments of eddies and to present the process of advection. The SST data used are provided by Group for High Resolution Sea Surface Temperature (GHRSST) Level 4 sea temperature analysis production based on a global 0.01 degree grid with the temporal resolution of 1 day. The Multiscale Ultrahigh Resolution (MUR) L4 analysis is based on SST observations from AMSRE, MODIS, WindSat, AVHRR and *in situ* observations from NASA.

Wind data used to calculate the nonlinear Ekman pumping combined with geostrophic currents. Wind data are the Cross-Calibrated Multi-Platform (CCMP) gridded surface vector winds which are produced by satellite, moored buoy, and model wind data. This daily production covers the global regions with the spatial resolution of 0.25°.

### Chlorophyll rings identification

**Figure 6:**
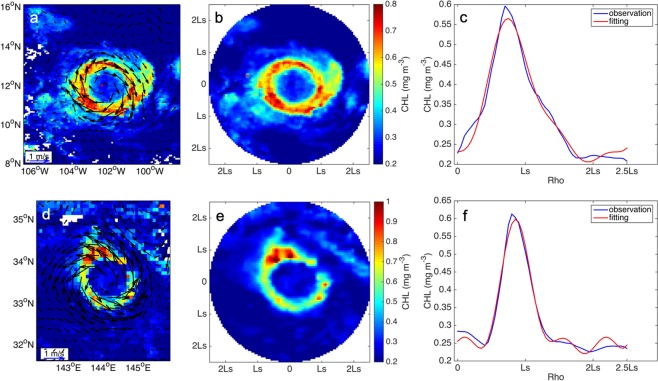
Identification of CRs. (**a**) An anticyclonic eddy (on Dec 31, 2008) matched with chlorophyll concentration data. The vectors are the velocity fields of sea surface. (**b**) Chlorophyll region is transformed into polar coordinates. (**c**) Distribution of the averaged chlorophyll as a function of the eddy radius. The peak of averaged chlorophyll concentration locates around Ls, which denotes the eddy radius. Rho refers to the normalized distance from the eddy center related to Ls. (**d**–**f**) same as (**a**–**c**) expect for a cyclonic eddy on Apr 27, 2005. Figures are plotted using MATLAB R2014b (http://www.mathworks.com) with M_Map (a mapping package, http://www.eos.ubc.ca/~rich/map.html).

Chlorophyll observations are linearly interpolated onto a normalized grid spanning +/−2.5Ls allowing missing data to be filled (Ls denotes the eddy radius). The normalized, eddy-centric chlorophyll concentrations are then transformed from a Cartesian grid (Fig. 6a) into polar coordinates (Fig. 6b). The average chlorophyll concentrations in different radial bins are calculated (Fig. 6c) and if the local extrema of averaged chlorophyll concentration are located between 0.5 Ls and 1.5 Ls, the feature is identified as a possible CR. In order to avoid that the chlorophyll concentration is extremely high locally, a similar process is applied to chlorophyll concentration along the 16 directions in the polar coordinates which are evenly distributed from 0° to 360°, checking whether the peak of chlorophyll concentration is between 0.5 Ls and 1.5 Ls in more than 9 directions. To all the possible rings, a threshold is applied to the range of chlorophyll between the maximum and minimum of 3 times of the minimum value. Figure 6d–f demonstrate the application of the method in a cyclonic eddy on Apr 27, 2005.

### Eddies identification

An automated algorithm based on the geometry of the velocity vectors of the flow field^31^ is used to identify and track mesoscale eddies based on the geostrophic current velocity which is calculated from the dataset of Merged Sea Level Anomalies (MSLA) acquired from AVISO. Eddy centers are determined by the spatial characters of the velocity fields, eddy sizes are computed from closed contours of the stream function field, and eddy tracks are retrieved by comparing the distribution of eddy centers at successive time steps.

Each eddy is matched with chlorophyll concentration data if it has a minimum lifetime of 4 weeks and the proportion of missing chlorophyll data do not exceed 70% for total number of pixels within an area defined as 2.5 time the radius scale of the eddy.
